# The Academic Self-Regulation Questionnaire: a study with Portuguese elementary school children

**DOI:** 10.1186/s41155-019-0124-5

**Published:** 2019-04-11

**Authors:** Marta Gomes, Vera Monteiro, Lourdes Mata, Francisco Peixoto, Natalie Santos, Cristina Sanches

**Affiliations:** 1 Department of Educational Psychology, ISPA – Instituto Universitário, Rua Jardim do Tabaco, 34, 1149-041 Lisbon, Portugal; 2 Centro de Investigação em Educação (CIE – ISPA), Lisbon, Portugal

**Keywords:** Self-determination theory, Motivation, Regulatory styles, Elementary school children, Mathematics

## Abstract

**Background:**

Several studies have focused on the assessment of students’ motivation because this construct is very important to understand students’ learning and how to enhance it. The Academic Self-Regulation Questionnaire (SRQ-A), based on the self-determination theory is a self-report instrument developed to access the reasons why students do their school work. However, there is no Portuguese version of this questionnaire for late elementary students. The primary goal of this research was to analyze the psychometric properties of a Portuguese version of SRQ-A in the domain of Mathematics with elementary school children.

**Methods:**

Participants were 341 elementary school children ranging from 8 to 11 years old from the third and fourth grades. The Portuguese version of the SRQ-A included 24 items assessing four regulatory styles (external, introjected, identified, and intrinsic) in three behavioral categories (homework, classwork, and answering questions in mathematics lessons). To examine the psychometric properties of the instrument, we conducted an exploratory structural equation modeling (ESEM), measured gender and grade invariance, and calculated internal consistency indexes and temporal stability.

**Results:**

ESEM analyses supported the original multidimensional structure of the measure with four regulatory styles using a reduced version of the instrument with 16 items. Correlations between the four regulatory styles revealed a simplex pattern consistent with the continuum of self-determination theory. Results showed adequate internal consistency for all regulatory styles (α ≥ .73; CR ≥ .76) and temporal stability (4-month test-retest ≥ .43). The questionnaire showed measurement and structural invariance across gender and grade. Finally, some gender differences were observed; on average, boys scored higher than girls in external regulation. No differences were observed between grades.

**Conclusions:**

Our findings suggest that the Portuguese version of the SRQ-A has good psychometric properties providing adequate support for its use in educational research on motivational styles, including studies concerning gender and grade differences in self-regulation.

**Electronic supplementary material:**

The online version of this article (10.1186/s41155-019-0124-5) contains supplementary material, which is available to authorized users.

## Background

Motivation is central to human action. Individuals differ in their level of interest, persistence, and engagement when performing different tasks. These differences may be observed between and within individuals across various situations and domains (Ryan & Deci, 2000a).

Self-determination theory (SDT, Deci & Ryan, 1985, 2000; Ryan & Deci, 2000a) addresses central questions concerning what people do and why do they do it. This theoretical framework provides definitions for intrinsic motivation and varied forms of extrinsic motivation, representing the dynamics of human motivation and the benefits and costs of different styles of behavioral regulation.

An action must be experienced as autonomous or self-determined to be intrinsically motivated, that is, volitional, free from pressures and external control. When people are intrinsically motivated, they perceive their behavior with an internal locus of causality, and they experience enjoyment and inherent interest. Extrinsic motivation has been commonly described as an impoverished form of motivation where actions are performed due to external constraints, that is, the presence of an operationally separable outcome, such as a reward or the avoidance of a punishment. Instead of considering extrinsic motivation as fundamentally opposed to intrinsic motivation, SDT goes beyond this typical distinction and details different motivational orientations based on the degree that they have been internalized (Ryan & Deci, 2000a). If intrinsic motivation is the prototype of self-determined activity, some forms of extrinsic motivation are more controlled while others are more autonomous (Deci & Ryan, 2000; Ryan & Deci, 2000b).

SDT describes a taxonomy of motivational types, arranged according to the degree to which behaviors are self-determined (i.e., emanate from the self). These different types of motivation can be arranged into a continuum of self-determination that specifies the corresponding regulatory styles used, and possible transitions between them (Deci & Ryan, 1985; Ryan & Connell, 1989). At one extreme of the continuum is *Amotivation*, the state of lacking any intention to act, a non-motivated and non-regulated behavior (Ryan, 1995). At the other extreme of the continuum is *Intrinsic Motivation*, which refers to performing an act with the highest degree of autonomy, on the basis of its inherent enjoyment and interest, being the expression of intrinsic regulation. This motivation form represents the prototypic category of truly self-determined (versus controlled) functioning. Between these two extremes are four forms of extrinsic motivation, organized by degree of autonomy or self-determination, which correspond to four regulatory styles (Ryan & Deci, 2000a).

The most basic form of extrinsic motivation is called *External Regulation*, where a person behaves under external pressures or contingencies administered by others. *Introjected Regulation* represents the second form of extrinsic motivation, where behaviors are sustained by cognitive-affective consequences, self-administered by individuals, through the dynamics of self and social approval (Assor, Vansteenkiste, & Kaplan, 2009; Ryan, 1995). The controller and the controlled are both aspects of the same individual, although regulations have not been yet assimilated into the self (Deci & Ryan, 1985). In this kind of regulation, which is not self-determined, individuals regulate their behaviors by anticipating self-attributed consequences, such as the threat of guilt and shame, or contingent self-worth (Deci & Ryan, 1985). The third regulatory style is *Identified Regulation*, a more autonomous form of extrinsic motivation. This regulation is adopted because of the identified personal value and importance of a particular activity. Identification will allow a more volitional behavior, but rather than being triggered by enjoyment, the behavior is instrumental (e.g., exercising faithfully for general health). The fourth and most autonomous form of extrinsic motivation is *Integrated Regulation*. When identified regulations are completely assimilated into one’s unified sense of self, integration is the natural outcome (Ryan, 1995). It represents complete congruence between individual needs and social values that are internalized. This regulatory style requires substantial maturation, self-awareness, and effort (Vansteenkiste, Niemiec, & Soenens, 2010). Actions become self-determined, sharing many qualities with intrinsic motivation; however, integrated regulation is still performed instrumentally, where the outcome is still separable from the behavior (Ryan & Deci, 2000a, 2000b).

Since school motivation is considered an essential element for academic success among psychologists, teachers, and parents, it appears pertinent to assess the relationship between educational outcomes and different types of motivation based on SDT (Ratelle, Guay, Vallerand, Larose, & Senécal, 2007). Some hypotheses, consistent with SDT, were verified empirically, mostly with samples of college and high school students. For example, the motivation of autonomously regulated students (via intrinsic and identified regulations) promotes various positive school outcomes, namely better academic performance (Bailey & Phillips, 2015; Gillet, Morin, & Reeve, 2017; Guay & Bureau, 2018; Kusurkar, Croiset, Galindo-Garré, & Ten Cate, 2013; Orsini, Binie, & Tricio, 2018; Renaud-Dubé, Guay, Talbot, Taylor, & Koestner, 2015; Richardson, Abraham, & Bond, 2012; Taylor et al., 2014), the use of deep study strategies (Kusurkar, Croiset, Galindo-Garré, & Ten Cate, 2013; Orsini et al., 2018), greater school persistence intentions (Ratelle et al., 2007; Renaud-Dubé et al., 2015), lower exhaustion from study (Kusurkar et al., 2013), and better academic adjustment (Boiché & Stephan, 2014; Ratelle et al., 2007). Autonomously regulated students also present better psychological well-being (Bailey & Phillips, 2015; Burton, Lydon, D’Alessandro, & Koestner, 2006), are more engaged in the learning process (Gillet, Morin, & Reeve, 2017; Oga-Baldwin, Nakata, Parker, & Ryan, 2017), and experience better educational satisfaction (Gillet et al., 2017), self-esteem, and vitality (Orsini et al., 2018).

Additionally, some studies have shown that higher levels of introjected and external regulation are negatively related to academic performance (Deci & Ryan, 2000; Taylor et al., 2014). However, Taylor et al. (2014) found that external regulation was positively associated with later achievement, and Ratelle et al. (2007) observed that students high in both autonomous and controlled motivations also have positive school achievement. Other studies have revealed a positive relationship between introjected regulation and persistence (e.g., Otis, Grouzet, & Pelletier, 2005; Renaud-Dubé et al., 2015). These results indicate that, in some cases, extrinsic and introjected motivation may have a positive effect on some academic outcomes. However, given the small number of studies with elementary school children, more research is needed.

Assessing SDT’s motivational constructs in the educational field requires rigorously designed instruments to advance motivational research and theory and generate practical applications. Among the most relevant instruments are the Academic Motivation Scale (AMS, developed by Vallerand, Blais, Brière, & Pelletier, 1989) and the Self-Regulation Questionnaire-Academic (SRQ-A, developed by Ryan & Connell, 1989). AMS was designed to assess motivation in post-secondary students while SRQ-A targets late-elementary and middle school students. The SRQ-A pertains to a set of questionnaires assessing the degree to which an individual’s motivation for a particular behavior tends to be relatively autonomous versus relatively controlled. It is based on self-reported reasons for engaging in school-related behaviors and contains four subscales that reflect the SDT continuum from extrinsically motivated to intrinsically motivated behaviors and the four corresponding regulatory styles: three forms of extrinsic motivation (external, introjected, and identified regulation) as well as intrinsic motivation (intrinsic regulation).

The psychometric properties of this questionnaire are described by Ryan and Connell (1989). The authors opted for a four-factorial structure (external, introjected, identified, and intrinsic) instead of a two-factor solution (internal and external) as a mean to account for the psychological meaningfulness of these categories. They confirmed that the regulatory styles were related to each other in a simplex-like pattern, which supports the self-determination continuum for motivational types. In a simplex pattern, variables are ordered in terms of conceptual similarity, where concepts that are more similar tend to correlate higher than theoretically more dissimilar concepts. Internal consistency for the four subscales showed moderate to high reliability, ranging from 0.62 to 0.82 (Ryan & Connell, 1989).

SRQ-A has been widely used, and its application has been extended to different countries and cultures. In Italy, with a sample of fourth grade students, Alivernini, Lucidi, and Manganelli (2011) replicated the four-factor structure that underlies the SRQ-A (15 items), the simplex pattern, and found high internal consistency. The Japanese version (Carreira, 2012) for fifth and sixth grades also showed acceptable internal reliabilities, but the analysis yielded three factors (16 items): intrinsic regulation, external regulation, and another factor, for both identified regulation and introjected regulation. Bağçeci and Kanadli (2014), with fifth to eight grade Turkish students, also replicated the SRQ-A structure (17 items), but external regulation presented low-level reliability. In a study with adolescents, Gnambs and Hanfstingl (2013) concluded that the German version of the SRQ-A (16 items) was a reliable instrument with no structural differences by gender or age. After that, Kroner, Goussios, Schaitz, Streb, and Sosic-Vasic (2017) replicated the simplex pattern of the German version (28 items) with a younger sample (third to sixth graders) but obtained a poor fit to the four-factor structure and found structural differences by gender. In general, these results have provided support for the validity and reliability of this questionnaire; however, they also highlight the need of some adjustments and research concerning its psychometric characteristics in different cultural contexts, ages, and domains (e.g., mathematics, language).

In the Portuguese context, there are few instruments based on SDT (Deci & Ryan, 1985, 2000; Ryan & Deci, 2000a), particularly within the academic context, whose psychometric properties have been examined with elementary school students (e.g., the Intrinsic Motivation Inventory, IMI, Monteiro, Mata, & Peixoto, 2015). As far as we know, there is not any Portuguese version of the SRQ-A for elementary students. Therefore, the main aim of this study was to translate the SRQ-A into Portuguese and to assess the psychometric properties of this Portuguese version with a sample of elementary school students. Specifically, we intended to test the fit of the data to the two alternative models proposed by Ryan and Connell (1989): the four-factor model (external, introjected, identified, and intrinsic) and the two-factor model (external and internal). Since motivation is domain specific (Chanal & Guay, 2015; Guay, Ratelle, & Chanal, 2008; Guay & Bureau, 2018), we tested the properties of the SRQ-A in the domain of mathematics, a core subject in school education that has high failure rates among Portuguese students (OECD, 2016).

Moreover, since previous studies reported inconsistent findings regarding gender and grade differences in the motivational orientations (see Barkoukis, Tsorbatzoudis, Grouios, & Sideridis, 2008; Guay et al., 2010; Kusurkar, Croiset, & Ten Cate, 2013; Vecchione, Alessandri, & Marsicano, 2014), we also examined these differences among Portuguese elementary school students concerning their regulatory style. Therefore, the factorial invariance of the structural model across gender and grades was tested to ensure the validity of these group comparisons.

## Method

### Participants

Data collected in this cross-sectional study were part of a broader longitudinal research project. Participants were 341 elementary school children ranging from 8 to 11 years old (*M* = 8.80, SD = 0.77, 47.8% female). Children attended the third (63.9%) and fourth grades (36.1%) and were from seven public and private schools in Lisbon and the surrounding areas, with diverse socioeconomic backgrounds.

### Procedures

Schools were contacted and asked to participate in the study. After receiving approval from the ethical committee (of the author’s research center), from the National Commission for Data Protection (CNPD), and from school boards, parental written consent was obtained before data collection. Confidentiality and anonymity were guaranteed, and the children’s participation was voluntary.

The questionnaire was administered in groups of five children, during regular school hours, in the exclusive presence of the researchers. Items were also read aloud by the researcher to ensure that all students, even those who could have reading or other learning difficulties, were able to understand what was being asked. Those students who were considered by their teachers as not being able to answer the questionnaire were not included in the study.

### Measures

The Portuguese version of the instrument was translated from the standard version of SRQ-A (based on Ryan & Connell, 1989; Self-Determination Theory, n.d.) and applied to the domain of mathematics. The items were translated using a back-translation procedure. This version was developed by an individual interview conducted by an expert interviewer. Some items were not well understood by third and fourth grade students because they contained linguistic expressions that are not typical in the Portuguese language. Therefore, those items were rephrased or altered, valuing a conceptual rather than literal translation (see SRQ-A in the Additional file 1). The modified items were evaluated by an expert according to the self-determination theory in order to ensure that they corresponded to the same regulatory style as the original items.

The same happened with the original wording of the response scale (1 = not at all true; 4 = very true), which was changed to the frequency scale used in the version of the SRQ-A developed for students with learning disabilities (Self-Determination Theory, n.d.). The response scale was also changed from the original four-point scale to a five-point scale (1 = never; 5 = always), since some studies indicated that this yields better quality data (e.g., Revilla, Saris, & Krosnick, 2013).

The version used in this study included 24 items representing three behavioral categories (each with eight items) that are central to academic performance: doing homework, doing classwork during the math lesson, and trying to answer questions in class. These behavioral categories were presented in the form of “why questions”: “Why do I do my math homework?”, “Why do I work on my classwork during math lessons?”, and “Why do I try to answer the questions in math lessons?” For each of these three categories, eight possible preselected reasons (items) were presented, representing four regulatory styles (external, introjected, identified, and intrinsic) with two items to assess each style (see SRQ-A in the Additional file 1). For example, the reasons representing each regulatory style for the question *Why do I do my math homework* were: *Because I do not want to be punished* (external); *Because I want the teacher to think I am a good student* (introjected); *Because it*’*s important to me to do my math homework* (identified); *Because I enjoy doing my math homework* (intrinsic).

### Data analysis

To examine the factor structure of the SRQ-A, we conducted an ESEM with the weighted least squares means and variance adjusted (WLSMV) estimator in MPlus 7.4, because of the categorical nature of the data (Barendse, Oort, & Timmerman, 2014). The two theoretical models proposed by Ryan and Connell (1989) were compared: in the four-factor model, factors were allowed to correlate, and the items were allowed to load freely on factors related to the four regulatory styles: external, introjected, identified, and intrinsic. In the two-factor model, factors were allowed to correlate, and the items were allowed to load freely on an external factor and an internal factor. Overall model fit was assessed using the following indices: chi-square (*X*^*2*^), comparative fit index (CFI), Tucker-Lewis index (TLI), root mean square error of approximation (RMSEA), and standardized root mean square residual (SRMR). Cut-off point recommendations of Schreiber (2017) were followed for goodness of fit indices criteria: CFI ≥ .95, TLI ≥ .96; RMSEA ≤ .05 (with confidence interval [.00, .08]); and SRMR ≥ .08.

Configural, metric, and scalar invariance was tested to investigate the potential presence of structural differences in the SRQ-A final model, based on participant gender and grade, as described by Xu and Tracey (2017). Differences between the models were examined with the chi-square difference test (Δ*X*^*2*^) and changes in CFI (ΔCFI), as recommended by Cheung and Rensvold (2002). In this regard, a non-significant result in chi-square difference test and ΔCFI lower than 0.01 in all comparisons indicate metric and scalar invariance (Xu & Tracey, 2017).

Means, standard deviations, and reliabilities (Cronbach’s alpha and composite reliability) for each of the four regulatory styles of the SRQ-A final model were calculated. Composite reliability (CR) was computed following the approach used by Colwell (2016). We calculated the test-retest correlation (4 months) using Pearson’s correlation coefficient with the sample of participants (*n* = 76) that were part of the longitudinal research project. Correlation analyses were conducted to test the expected simplex pattern of the regulatory styles.

## Results

The goodness-of-fit of the two alternative models is reported in Table 1.

**Table 1:** Goodness of fit indexes for the proposed models

Model	*X* ^2^	*df*	CFI	TLI	RMSEA	SRMS
Two-factor model	597.39	229	.952	.943	.069	.048
Four-factor model	339.86	186	.980	.970	.049	.032
Four-factor model re-specified	4915.38	120	.984	.969	.060	.025

*n* = 341

These results showed that the fit values of TLI and RMSEA for the two-factor model did not meet the standards that are currently recommended (Schreiber, 2017; the two-factor model estimates can be observed in the Additional file 2 - Table 1). The four-factor model presented a better solution (higher CFI and TLI; and lower *X*^2^, RMSEA, and SRMS), with the fit values meeting all of the standards. However, item 24 did not load on its corresponding factor and there were several cross-loadings (items 4, 7, 12, 16, 19, 20, and 21). The four-factor model estimates can be observed in the Additional file 2 - Table 2. Therefore, we re-specified the four-factor model, excluding these eight items with loading problems, since the remaining 16 items still represent the four regulatory styles proposed by Ryan and Connell (1989), with four items each.

**Table 2:** Factor loadings and correlations for the four-factor model re-specified (16 items)

Factor	Item	1	2	3	4
1. External	2	.*752*	.086	.141	.17
	6	.*799*	.042	.117	.052
	9	.*730*	.079	.042	.036
	14	.*785*	.144	.025	.090
2. Introjected	1	.036	.*705*	.106	.062
	10	.021	.*651*	.141	.038
	17	.178	.*648*	.060	.013
	18	.178	.*647*	.168	.068
3. Identified	5	.047	.036	.*840*	.015
	8	.024	.037	.*644*	.149
	11	.065	.167	.*645*	.106
	23	.025	.084	.*625*	.114
4. Intrinsic	3	.013	.012	.155	.*724*
	13	.172	.062	.033	.*969*
	15	.036	.159	.*327*	.*502*
	22	.150	.293	.085	.*674*
Factor correlation	2. Introjected	.*57*			
	3. Identified	.08	.*31*		
	4. Intrinsic	.06	.18	.*66*	

*n* = 341; loadings and correlations with *p* < .001 are in italic

The re-specified four-factor model with 16 items presented a good solution with the fit values meeting all of the standards, except for the RMSEA fit value. Yet, the confidence interval of the RMSEA is between .047 and .074, which is adequate.

All trajectories were statistically significant, and all items had λ ≥ .50 (Table 2). Only item 15 presented a cross-loading, but the item still loads strongly on its respective factor. For this final model with 16 items, measurement invariance held for all comparisons by gender and grade (see Table 3).

**Table 3:** Fit indices for invariance test of the four-factor model (16 items)

Model	*X* ^2^	*df*	*p*	CFI	RMSEA	*ΔX* ^2^_*a*_	*p*	ΔCFI
Configural invariance for gender	327	196	< .001	.973	.063			
Metric invariance for gender	333.3	208	< .001	.974	.059	6.98	.859	− .001
Scalar invariance for gender	371.5	252	< .001	.975	.053	39.04	.684	− .002
Configural invariance for grade	349.5	196	< .001	.969	.068			
Metric invariance for grade	353.8	208	< .001	.971	.064	9.0	.702	− .002
Scalar invariance for grade	392.1	252	< .001	.972	.057	39.96	.645	− .001

*n*_male_ = 178; *n*_female_ = 163; *n*_3rd_ = 218; *n*_4th_ = 123; *a* = the chi-square value for WLSMV cannot be used for chi-square difference testing in the regular way as described on the Mplus website. WLSMV difference testing was done using the DIFFTEST option

The expected simplex pattern of the SRQ-A emerged in four-factor model with 16 items (Table 2): external regulation had a higher correlation with the regulatory style deemed more similar (introjected), and lower correlation with the regulatory styles that were theoretically dissimilar (identified and intrinsic). The same pattern was observed for intrinsic regulation. Composite reliability for each factor showed good evidence of internal consistency (Table 2).

Mean, standard deviations, Cronbach’s alphas, and correlations between the four regulatory styles of the SRQ-A are presented in Table 4. Cronbach’s alphas for all regulatory styles showed satisfactory reliability. Four-month test-retest correlations indicated that introjected regulation was the least stable style, and intrinsic regulation was the most stable.

**Table 4:** General psychometric characteristics of the Portuguese SRQ-A (16 items)

SRQ-A	CR	α	Test-Retest^a^	Mean	SD	External	Introjected	Identified
External	.85	.80	.53	3.3	1.2	1		
Introjected	.76	.73	.43	3.1	1.0	.*54*	1	
Identified	.79	.75	.53	4.2	0.8	.07	.*21*	1
Intrinsic	.82	.86	.61	3.6	1.0	.04	.*24*	.*64*

*n* = 341; *CR* composite reliability; correlations with *p* < .001 are in italic

^a^*n* = 76

There were mean differences between the four regulatory styles (*F* (2.067,702.639) = 105.40, *p* < .001, η^2^_p_ .24, π = 1.00). Pairwise comparisons (with Bonferroni adjustment) indicated that all scores were significantly different (*p* < .001). Participants scored higher in the identified regulation style, and lower in the introjected regulation style. Correlations between the four regulatory styles appeared to conform to the simplex pattern.

Girls and boys significantly differed only in terms of external regulation, with boys scoring higher on average than girls (Table 5). However, these gender differences had a very small effect size. There were no differences between regulatory styles in third and fourth graders.

**Table 5:** Mean and standard deviation by gender and grade

SRQ-A	Gender					Grade				
	Male	Female	*T*	*p*	*d*	3rd Grade	4th Grade	*T*	*p*	*d*
	*M* (*SD*)	*M* (*SD*)				*M* (*SD*)	*M* (*SD*)			
External	3.5 (1.2)	3.2 (1.2)	2.38	.018	.13	3.4 (1.2)	3.2 (1.2)	1.46	.146	.08
Introjected	3.2 (1.0)	3.0 (1.0)	1.50	.135	.08	3.2 (1.0)	3.0 (1.0)	1.59	.114	.09
Identified	4.2 (0.8)	4.3 (0.7)	− 0.88	.377	.05	4.2 (0.8)	4.3 (0.6)	− 1.24	.215	.07
Intrinsic	3.6 (1.0)	3.6 (1.0)	− 0.12	.902	.01	3.7 (1.0)	3.6 (1.0)	0.74	.460	.04

Sample: *n*_male_ = 178; *n*_female_ = 163; *n*_3rd_ = 218; *n*_4th_ = 123

## Discussion

The main goal of this study was to evaluate the psychometric properties of the Portuguese version of the SRQ-A for elementary school children as a self-report measure for regulatory styles in mathematics. The results of this study replicated the four-factor model of the original SRQ-A (Ryan & Connell, 1989) and showed it was well suited to explain the Portuguese data. The 16 items in the Portuguese version demonstrated significant loadings on their expected latent factor. Additionally, correlations between the four regulatory styles revealed a simplex pattern consistent with the continuum of self-determination observed in several studies (Alivernini et al., 2011; Howard, Gagné, & Bureau, 2017; Kroner et al., 2017; Ryan & Connell, 1989).

Acceptable levels of internal consistency for all regulatory styles were obtained, comparable to the original version of the SRQ-A (Ryan & Connell, 1989). The values of temporal stability (test-retest) were lower than those found by Barkoukis et al. (2008) and Vallerand et al. (1992). However, both studies had only 1-month test-retest. Lower correlations would be expected in a 4-month test-retest because, as mentioned in the original scale description, regulatory styles vary by time and place (Self-Determination Theory, n.d.).

Overall, identified regulation scored higher on average (identified > intrinsic > external > introjected). This finding seems to show that children from our sample had a good understanding of the personal value and relevance of math activities for themselves, using this understanding to regulate their behavior.

Consistent with other validation studies, the test for model invariance revealed scalar invariances in self-regulation on mathematics between boys and girls (Alivernini et al., 2011; Grouzet, Otis, & Pelletier, 2006; Howard et al., 2017). This result provides some evidence for the applicability of the SRQ-A in studies concerned with gender differences in self-regulation. Significant differences between boys and girls were observed in external regulation, but the effect size was small. Our results are similar to those of Grouzet et al. (2006) and Ratelle et al. (2007) with older samples, and also those of Vecchione et al. (2014) with an elementary school sample (fourth and fifth grade), showing boys as more controlled and externally regulated than girls. However, as aforementioned, some studies have reported conflicting results, such as girls showing more intrinsic motivation than boys (Barkoukis et al., 2008; Kroner et al., 2017; Vallerand et al. ,^1992^), and no observable significant differences (Deci, Hodges, Pierson, & Tomassone, 1992). It is important to mention that in most of these studies, effect sizes were either small or not reported. Only Vecchione et al. (2014) found moderate effects, bringing into question whether gender differences have implications for research and practice. Further studies are needed to clarify these differences.

Some limitations should be considered in the present study as they may provide fruitful directions for future research. As the SRQ-A is a self-report questionnaire, it is vulnerable to various distortions in self-perception. Future studies should include teacher and parent reports, which might provide more comprehensive insight into student regulatory styles. As research with older students has demonstrated that regulatory styles are domain-specific, it may be necessary to extend these results by exploring whether SRQ-A is an appropriate measure for elementary students’ regulatory styles in other academic domains. It would also be interesting to carry out predictive validity studies with the Portuguese version of the SRQ-A, and to test, for example, whether differences in students’ regulatory styles are predictive of several academic outcomes such as effort, persistence, learning strategies, and performance. Since our data collection was cross sectional, a longitudinal approach would be useful to further understand the change and stability of motivational orientations towards mathematics during elementary school.

## Conclusions

Our study supported that the Portuguese version of the SRQ-A for mathematics has good psychometric properties and can be a useful tool both for research and for educational interventions at the elementary school level. Recently, several studies have already successfully used the SRQ-A to test the effectiveness of different intervention programs designed to improve intrinsic motivation (Bolling, Otte, Elsborg, Nielsen, & Bentsen, 2018; Vennix, den Brok, & Taconis, 2018). In addition, this contribution could have practical implications, allowing more focused interventions based on the understanding of motivational orientations proposed by SDT. For example, for students with low levels of identified and intrinsic regulation, it is important to communicate the value of doing uninteresting activities to provide rationales to engage in the requested behaviors (Niemiec & Ryan, 2009). For students with high levels of introjected regulation, it is important to create an environment of emotional support where students feel safe to express their feelings, doubts, and questions. In this way, they will not feel the need to seek out social approval, but instead develop a feeling of “relatedness” with the teacher and their classmates, which in turn would support the enhancement of intrinsic motivation (Guimarães & Boruchovitch, 2004; Niemiec & Ryan, 2009; Ryan & Deci, 2000a). Thus, the current study provides a suitable instrument to assess a continuum of self-determination, which is essential in understanding motivational regulations that reflect the various motives behind children’s engagement in mathematics.

## Additional files

Additional file 1: Items of the Portuguese version of SRQ-A. (DOCX 16 kb)

Additional file 2: Two-Factor Model and Four-Factor Model. (DOCX 16 kb)

## Abbreviations

AMS: Academic Motivation Scale
CFI: Comparative fit index
CR: Composite reliability
RMSEA: Root mean square error of approximation
SDT: Self-determination theory
SRQ-A: Academic Self-Regulation Questionnaire
TLI: Tucker-Lewis index
