# Supplementary material for: The Academic Self-Regulation Questionnaire: a study with Portuguese elementary school children
Source: Psicol Reflex Crit. 2019 Apr 11;32:8. doi: 10.1186/s41155-019-0124-5 (PMC6966964; doi:10.1186/s41155-019-0124-5)
Supplement: Supplementary file 1 — Items of the Portuguese version of SRQ-A. (DOCX 16 kb) [file 41155_2019_124_MOESM1_ESM.docx]

# Additional File 1.

# Items of the Portuguese Version of SRQ-A

# Why I do things

1. **Why do I do my math homework?**
2. **I do my math homework because I want the teacher to think I’m a good student**
3. **I do my math homework so that my parents and teacher won’t be mad at me**^1^ (original: because I’ll get in trouble if I don’t)
4. **I do my math homework because it’s fun**
5. I do my math homework because I will feel ashamed if I don’t do it^1^ (original: because I will feel bad about myself if I don’t do it)^2^
6. **I do my math homework because I want to understand the subject**
7. **I do my math homework so that I won’t be punished^1^** (original: because that’s what I’m supposed to do)
8. I do my math homework because I enjoy doing my homework^2^
9. **I do my math homework because it’s important to me to do my homework**
10. **Why do I work on my classwork during math lessons?**
11. **I work on my math classwork so that the teacher won’t scold me**^1^ (original: so that the teacher won’t yell at me).
12. **I work on my math classwork because I want the teacher to think I’m a good student**
13. **I work on my math classwork because it’s important to learn new things**^1^ (original: because I want to learn new things)
14. I work on my math classwork so that others don’t think badly of me^1^ (original: because I’ll be ashamed of myself if it didn’t get done) ^2^
15. **I work on my math classwork because it’s fun**
16. **I work on my math classwork because the teacher tells me to do it**^1^ (original: because that’s the rule)
17. **I work on my math classwork because I enjoy the tasks that are done there**^1^ (original: because I enjoy doing my classwork)
18. I work on my math classwork because it’s important for me to do well^2^
19. **Why do I try to answer questions in class?**
20. **I try to answer questions in math class because I want the other students to think I’m smart**
21. **I try to answer questions in math class so that I feel I am better than the others**^1^ (original: because I feel ashamed of myself when I don’t try)
22. I try to answer questions in math class because I enjoy math^2^
23. I try to answer questions in math class because that’s what the teacher wants^1^ (original: because that’s what I’m supposed to do) ^2^
24. I try to answer questions in math class to find out if I’m right or wrong^2^
25. **I try to answer questions in math class because it’s fun**
26. **I try to answer questions in math class because it’s important for me to try to do it**
27. I try to answer questions in math class because I want the teacher to say nice things about me^2^

^1^ Item rephrased or altered.

^2^ Item excluded in the final version with 16 items.
