# Supplementary material for: The Academic Self-Regulation Questionnaire: a study with Portuguese elementary school children
Source: Psicol Reflex Crit. 2019 Apr 11;32:8. doi: 10.1186/s41155-019-0124-5 (PMC6966964; doi:10.1186/s41155-019-0124-5)
Supplement: Supplementary file 2 — Two-Factor Model and Four-Factor Model. (DOCX 16 kb) [file 41155_2019_124_MOESM2_ESM.docx]

# Additional File 2.

Table S1
*Factor loadings (with p < .050) for the two-factor model*

|  |  | Factor loadings | |
| --- | --- | --- | --- |
| Factor | Items | 1. External | 2. Internal |
| 1. External | 2 |  | .797 |
|  | 6 |  | .811 |
|  | 9 | -.151 | .758 |
|  | 14 | -.129 | .628 |
|  | 20 |  | .552 |
|  | 24 |  | .722 |
|  | 1 | .168 | .595 |
|  | 4 |  | .546 |
|  | 10 | .275 | .552 |
|  | 12 |  | .767 |
|  | 17 |  | .720 |
|  | 18 |  | .697 |
| 2. Internal | 5 | .705 |  |
|  | 8 | .720 | .102 |
|  | 11 | .633 |  |
|  | 16 | .457 | .177 |
|  | 21 | .431 | .156 |
|  | 23 | .663 | .172 |
|  | 3 | .837 |  |
|  | 7 | .853 | -.089 |
|  | 13 | .827 |  |
|  | 15 | .784 | .090 |
|  | 19 | .861 | -.151 |
|  | 22 | .800 |  |

*Note.* n = 341

Table S2
*Factor loadings (with p < .050) for the four-factor model*

|  |  | Factor loadings | | | |
| --- | --- | --- | --- | --- | --- |
| Factor | Items | 1. External | 2. Introjected | 3. Identified | 4. Intrinsic |
| 1. External | 2 | .652 |  |  |  |
|  | 6 | .680 |  |  |  |
|  | 9 | .629 |  |  |  |
|  | 14 | .665 |  |  |  |
|  | 20 | .233 | .349 | .285 |  |
|  | 24 |  | .722 |  |  |
| 2. Introjected | 1 |  | .664 |  |  |
|  | 4 | .340 | .279 |  |  |
|  | 10 |  | .62 |  |  |
|  | 12 | .391 | .529 |  |  |
|  | 17 |  | .793 |  |  |
|  | 18 |  | .802 | .207 |  |
| 3. Identified | 5 |  |  | .777 |  |
|  | 8 |  |  | .669 |  |
|  | 11 |  |  | .655 |  |
|  | 16 | .250 |  | .509 |  |
|  | 21 |  | .231 | .448 |  |
|  | 23 |  |  | .659 |  |
| 4. Intrinsic | 3 |  |  | .137 | 0.769 |
|  | 7 | .151 |  | .407 | 0.489 |
|  | 13 | .276 |  |  | 0.949 |
|  | 15 |  | .152 | .302 | 0.526 |
|  | 19 | .170 |  | .392 | 0.513 |
|  | 22 |  | .217 |  | 0.731 |

*Note.* n = 341
